# A Framework for Quantifying the Strength of Partnerships between Agricultural Cooperatives and Development Actors: A Case Study in Saudi Arabia

**DOI:** 10.3390/ijerph20010364

**Published:** 2022-12-26

**Authors:** Ahmed Herab, Ahmad Al-Ghamdi, Khodran Alzahrani, Khalid M. Elhindi, Muhammad Muddassir, Hazem S. Kassem

**Affiliations:** 1 Department of Agricultural Extension and Rural Society, College of Food and Agriculture Sciences, King Saud University, Riyadh 11451, Saudi Arabia; 2 Department of Plant Protection, College of Food and Agriculture Sciences, King Saud University, Riyadh 11451, Saudi Arabia; 3 Department of Plant Production, College of Food and Agriculture Sciences, King Saud University, Riyadh 11451, Saudi Arabia; 4 Department of Agricultural Extension and Rural Society, Faculty of Agriculture, Mansoura University, Mansoura 35516, Egypt

**Keywords:** beekeeping, cooperatives, non-profit organizations, partnerships, rural development, sustainability

## Abstract

The issue of the agricultural cooperatives’ sustainability in developing their businesses is gaining increasing prominence. Building partnerships between development actors and agricultural cooperatives has been considered an effective strategy for supporting financial capital and addressing sustainability issues collectively. Therefore, this study aimed to address the features and strengths of 33 partnerships established between the Beekeeping Cooperative Association in Al-Baha, Saudi Arabia, and other actors between 2016 and 2021. The analysis of the collaborations was based on six criteria: motivations, partnership planning, outputs, governance practices, outcomes, and sustainability of a partnership. Furthermore, we developed a weighted scoring model to control variable selection and submit the strength of each partnership. The findings indicated that most collaborations (45.5%) were signed with the private sector. Furthermore, the honey value chain development was the most frequent reason (69.7%) attracting the partners to engage in the partnerships. Some of the most critical environmental objectives targeted by the partnerships examined were enhancing bee habitat by the diversification of pasture species, management to increase the flowering period, and proper grazing management. All partners achieved their individual goals jointly in 54.5% of the partnerships analyzed. In terms of a partnership’s strength, the findings also revealed that only three partnerships (9.1%) were characterized as strong partnerships. This study provides a better understanding of how agricultural cooperatives collaborate with other actors and a basis for assessing the strength of the partnerships. Such information is crucial for developing relevant policies to encourage cooperatives to engage in future sustainability partnerships.

## 1. Introduction

Developing and strengthening partnerships between public and private organizations is highlighted by the United Nations’ Sustainable Development Goals (SDGs) (SDG-17: Partnerships for the goals) [1]. The peculiarity of partnerships in achieving the SDGs is that pursuing common interest objectives includes capital, expertise, or information. These factors are difficult or costly to address individually, often due to a lack of resources [2,3]. Furthermore, partnerships are vital for organizations to develop innovative and cost-effective solutions for complex development issues [4]. These issues are multifaceted and multilayered, marked by critical tradeoffs between risks, costs, outcomes, and externalities [5,6]. Given the complexity and scale of sustainable development challenges, developing multi-stakeholder partnerships with a broad range of interests is beneficial to increasing the likelihood of a successful partnership and supporting the organization’s capacity to solve these challenges in all sectors [7,8].

Among various organizations in the agricultural sector, agricultural cooperatives play a leading role in achieving SDGs substantially on a global level [9,10]. The enormous contribution of cooperatives may be noticed in the efforts to address the challenges posed by transitions to sustainability. These efforts include improving food security, natural resource management, poverty alleviation, gender equality, and promoting life-long learning opportunities [10,11]. International development organizations, such as the International Co-operative Alliance, the International Labour Organization, and the United Nations have argued that the cooperative model is suited well to address sustainable development challenges [11]. Three main areas of thought support this argument. First, the cooperative values and principles are consistent and harmonious with the SDGs and their indicators [12]. Second, cooperatives often meet social, economic, and environmental goals simultaneously [13]. Third, the cooperatives also apply good governance practices by facilitating education and training, enabling people to assume their own development, solve common problems, and foster member economic participation [10]. Therefore, despite the compatibility of cooperatives in tackling sustainability issues, insufficient resources disable cooperatives from effectively addressing several challenges in the agriculture sector. These challenges include innovation, digitalization, the COVID-19 pandemic, food safety and traceability, climate change and natural resource management, marketing, product value addition, value chain development, farm input issues, governance and gender issues, and human resource development [9,13]. Therefore, cooperatives have adopted new business models with multiple civil, public, and private actors at local and international levels to support the transformation process [14,15].

Adopting transformation processes by the cooperatives requires enabling a conducive internal and external environment to build successful partnerships [16]. Nonetheless, internal factors stimulate partners to work collaboratively for mutual benefit. However, the cooperative capacity of the partnerships influences the strength of the partnership [17]. According to Vayaliparampil, Page and Wolterstorff [4], the cooperative capacity is conceptualized by nine elements: balance between costs and benefits, self-management, fast and fair conflict resolution, fair and inclusive decision-making, group identity and understanding of purpose, graduated sanctions, monitoring behaviors and performance, minimal recognition by stakeholders, and the ability to adapt. Franke et al. [18] noted that these elements reflect three conditions. The first includes sharing governance roles and responsibilities between partners equitably. The second covers a common understanding of the roles, processes, and practices in the partnership’s governance. The third addresses partners’ trust in each other’s words, decisions, and actions regarding the partnership. Consequently, building a successful partnership demands carefully analyzing all the elements affecting value optimization [19]. Nevertheless, these elements are varied and interrelated depending on the specific context and the partners involved in the partnership [20]. Regardless, external factors (sociopolitical) create and influence the conditions for collaborations to occur. In 2016, Saudi Arabia issued the 2030 Vision, where the objective strategy of “empowering the cooperative sector by building partnerships” is one of the primary strategic objectives in the transformation plan to achieve the aims and objectives of this vision [21]. In this context, the study of Kassem, Aljuaid, Alotaibi and Ghozy [19] confirmed the importance of Saudi Vision 2030 as a driver for growth opportunities for collaboration. In their analysis, they also mentioned the drivers of partnerships in the non-profit sector as high interest among private companies to collaborate with the associations as a part of their plan to achieve corporate social responsibility.

To the best of our knowledge, no research has either addressed the extent of the strength of the agricultural cooperatives–other actors’ partnerships or how features of strength may influence achieving SDGs by the cooperatives. Therefore, this study presents a new model to measure the strength of partnerships. More comprehensively, it supplies a holistic framework describing the fundamental components for successfully building partnerships. This original model is derived and developed based on previous papers of Hazem S. Kassem and his team [17,19,22]. These authors analyzed the nature of the partnerships built by non-profit organizations with other sectors. Our article suggests this model as a sense-making construct guide for an in-depth analysis of partnerships between the beekeepers’ cooperative associations in Al-Baha, Saudi Arabia, and other public and private actors. It also presents insights that could optimize the model’s utility for characterizing and governing the partnerships.

## 2. Theoretical Framework

Globally, agricultural cooperatives have faced international development challenges. These challenges cannot be overcome separately. They may be due to allocating resources and responsibilities to a wide range of public and private actors and the complicated nature of some agricultural problems [23,24]. Hence, forming successful partnerships between cooperatives and public and private actors can be an effective strategy for cooperative sustainability and services for rural development. However, building a solid partnership is a complex process. It requires specific features in the transformation processes to facilitate collaboration and engage various stakeholders effectively [25,26]. These transformations are essential to the broader political, economic, social, and organizational contexts encouraging collaborations [27]. This study focuses on the internal environment by addressing the organizational components determining a solid collaboration. Details of these components are provided below.

### 2.1. Motivations

Motivations are internal factors creating the conditions for partnership formation [28]. They are part of the organizational context, and this context promotes partnership formation [29]. The literature has identified various organizational factors that facilitate building partnerships, including legitimacy, resources, competencies, and society-oriented motivations [30]. Legitimacy refers to social acceptance; a partnership meets societal norms and expectations [28]. Furthermore, partners may have broader resources-oriented motivations for collaboration [29]. Various resources that attract partners for collaboration include training programs, gaining goods and services, funding the activities of the organization’s strategic plan, and innovation in production and marketing [30,31]. Regarding competencies, gaining new knowledge and enhancing skills motivate partnership engagement [19]. In this sense, Gray and Stites [29] clarified that the differences in knowledge, skills, and capabilities between partners stimulate acquiring multiple areas of expertise. Finally, the importance of partnerships for society cannot be ignored as a critical motivation for collaboration. The society-oriented motivations are complex and involve a diverse range of social, economic, and environmental issues, such as community mobilization, policy development, responding to social and environmental problems, and raising public awareness of international development issues [32,33]. In this context, Gray and Purdy [34] argued that such motivations promote community mobilization and strengthen society’s capacity to solve sustainability issues.

### 2.2. Planning of a Partnership

A well-defined partnership plan is critical for partnership sustainability [19]. Analyzing partnership planning requires a deep understanding of partnership configuration concerning partners, timescale, legal form, geographical coverage, purpose, objectives, and typologies of a partnership [25].

#### 2.2.1. Partner

Finding a suitable partner is challenging due to ideological differences between agricultural cooperatives and other actors [35]. Selecting the right partner suited to implement a partnership is linked to several factors, such as the partnership’s objectives, nature of activities, and partnership typologies [33]. Therefore, building trust-based collaboration between agricultural cooperatives and other actors needs to consider four areas to identify the right counterpart: solid criteria for selection, institutional form of a partnership (Intra-sector or inter-sector), nationality of partners, and the sectors that the partner is belonged [17]. 

#### 2.2.2. Legal Form

Once both partners have accepted the partnership proposal, the partnership can be classified as a formal contractual arrangement with the exchange of funds or informal, minimal work between parties [36]. However, the formal form of a partnership is the only way for governance structure promotion to resolve internal conflicts, manage the evolution of the partnership, and provide direction [37]. In this sense, Afansa, et al. [38] listed four formal arrangements between parties: a simple written document defining the terms of a partnership (letter of association), a written agreement between partners to establish objectives, roles, and responsibilities (Memorandum of Understanding (MoUs)), Terms of Reference (TOR), and an agreement between partners, usually enforceable by law (contract). 

#### 2.2.3. Timeframe

Regarding the timeframes of the collaborations, partnerships are short-term or long-term projects. The limited duration of collaborations leads to discontinued and interrupted relationships [22]. However, Manning and Roessler [39] have a different view suggesting that some short-term partnerships are designed to transform relationships in the long term. On the contrary, long-term partnerships are a positive prerequisite for sustaining outcomes [40]. 

#### 2.2.4. Geographical Coverage

Regarding geographical coverage, agricultural cooperatives–other partners’ partnerships may be implemented at varying levels (village, city, governorate, region, national, or international) based on the available fund, stakeholders, and partnership’s objectives [41]. 

#### 2.2.5. Purpose

The literature highlighted many purposes enacted by the cooperatives during collaboration, including food security, capacity building, access resources, innovation and technology transfer, market infrastructure development, and value chain development [42,43]. 

#### 2.2.6. Objectives

Partners’ objectives differ according to the area of interest between partners, problems, needs, and priorities of the stakeholders, typologies of the partnership, specialization, capabilities, and strategic plan of the cooperatives [16,24]. In agricultural cooperatives, the objectives cover a wide range of social, economic, and environmental issues. Social services to the partnership’s target people include subsidized inputs, direct funds, indirect funds, in-kind funds, and recruitment [44,45]. Nonetheless, economic services focus on increasing productivity, enhancing quality, and marketing [46]. Additionally, agricultural cooperatives–other actors’ partnerships may involve valuable environmental services for community sustainability. These services cover environmental education, pollution control, waste management, influence or pending or averting imminent environmental regulations, land protection and rehabilitation, and promoting activities of agri-tourism [13,47,48,49].

#### 2.2.7. Types of Partnerships

In recent years, various types of partnerships have been observed in collaborations between agricultural cooperatives and private and public actors. The principle of no “one size fits all”—context matters encourages the adoption of different types of partnership typologies [43]. This study depended upon the classification of Austin [50] for types of partnerships according to the degree of business versus social orientation. According to this classification, two forms of partnerships exist: transactional and strategic. Transactional partnerships are labeled as “philanthropic partnerships”, where financial assets, services, products, workforce, or other resources transfer one way as donations from one partner to others. However, they could also be characterized as “social investments”, where resources can be used strategically by engaging in a mutual or reciprocal exchange of activities and services. Thus, two types of strategic partnership can be distinguished: (1) new commercial initiative partnerships, where all partners collectively attempt to develop individual value creation by matching their individual goals, and (2) core-business partnerships or integrative partnerships, where all partners work jointly to address a problem, develop a business, or a new service in which all partners receive a mutual benefit.

### 2.3. Governance

Partnership governance has received growing academic interest due to the differences between cooperatives and other public or private actors in their objectives and configurations [51]. Therefore, good governance practices are required to define decision-making processes, roles and responsibilities, accountability, and managing risk before entering into a partnership [52,53]. 

#### 2.3.1. Managing and Maintaining Practices

Understanding managing and maintaining practices and their interactions is crucial to enable partners to set the partnership’s direction [54]. The managing and maintaining phase is to allocate the roles and responsibilities between partners for service delivery or program as planned, jointly manage and monitor the activities, and keep parties informed of their progress toward achieving planned objectives [55]. 

#### 2.3.2. Revising and Reviewing Practices 

In this phase, partners assess the outcomes of the partnership to determine its success. Partners in this phase implement two steps: review the value of the partnership and suggest the improvements in terms of specifying needs to be changed, providing a timetable for change management process, and applying the agreed-upon changes [56].

### 2.4. Outputs

#### 2.4.1. Activities and Services

Partnership outputs represent products resulting from activities. These products are classified into goods (tangible or visible items) and services (invisible or intangible items). The delivery of products is the primary indicator describing the outputs. In the M&E plan, partners should suggest output indicators to describe the delivery of products. These indicators include investing in buildings and infrastructure, creating standards and legislative documents, providing training and technical assistance, and hiring staff required to implement a partnership [57]. 

#### 2.4.2. Stakeholders

Analyzing partnership outputs include types of beneficiaries from the products delivered. In cooperatives, partnerships established between agricultural cooperatives and other actors mainly target the members of cooperatives [58]. Some partnerships or programs within the same partnership may extend their target people to include a wide range of farmers in various fields, poor or low-income farmers, youth, rural women, or people with disabilities [17]. Determining the target people list accurately depends on the partnership’s objectives, the types of goods and services provided, geographical coverage, and the cooperative’s specialization [10].

### 2.5. Outcomes

Outcomes are the changes expected to result from a partnership in the short, medium, and long terms [59]. Measuring intended outcomes is essential to determining partnership effectiveness [4]. Austin and Seitanidi [60] argued that managing partnerships to achieve meaningful outcomes is required for monitoring resources, supporting the public accountability of a partnership, and concluding lessons learned. 

#### 2.5.1. Benefits

The partnerships’ benefits vary according to the context, stakeholders’ needs, and partner expectations [39]. Undoubtedly, acquiring resources (including technical and managerial expertise, volunteers, goods, services, and investments) is crucial in attracting partners for collaboration [29]. In the same vein, partnerships also target outcomes such as environmental protection, organizational innovation, effective services, better access to information, marketing, and human capital development [59].

#### 2.5.2. Impact Assessment

The outcomes would accurately reflect the success of partnerships if it assessed professionally by adopting evaluation tools and methodologies [61]. Therefore, outcome measurement should be constantly addressed in monitoring and evaluating a partnership’s M&E plan [43].

### 2.6. Partnership Sustainability

#### 2.6.1. Sustainability Plan 

Sustaining outcomes is a leading indicator of partnership sustainability. The first step in suggesting a sustainability plan for a partnership is to scale it up to benefit more people and increase the impact and influence if the partnership is successful [22]. Pfisterer [62] suggested some activities be accomplished in the scaling step, such as publicizing outcomes using information communication technologies and expanding the signed partnerships. In the final step of the partnership life-cycle, partners decide whether to continue. This step involves many options, including continuing to work jointly on new or the same projects, working alone or with new partners, or developing core-business enterprises based on the resources of the partnership [63]. In this context, KPMG [64] noted that depending upon the partnership’s outcomes, partners might decide to continue the partnership or end it. 

#### 2.6.2. Current Situation of a Partnership 

Determining the continuum of the partnership’s sustainability is crucial for summarizing lessons learned and enriching sustainability plan for the continued partnerships. The current situation of a partnership could be involved various forms, including renew the partnership annually, completion of the partnership and all objectives accomplished, completion of the partnership and objectives partially accomplished, completion of the partnership and objectives not achieved, or contract termination [19].

**Figure 1:**
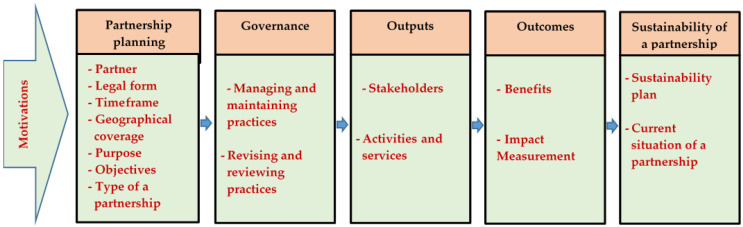
Conceptual framework of the study.

Based on the literature review, this paper devised a conceptual framework to achieve the objective (Figure 1). This framework involved six components: motivations, planning of a partnership, governance practices, outputs, outcomes, and sustainability of a partnership. These components were grouped into 15 sub-components. They covered partner, legal form, time scale, geographical coverage, the purpose of a partnership, objectives of a partnership, and type of a partnership, stakeholders, services provided by a partnership, managing and maintaining practices, reviewing and revising practices, benefits gained from a partnership, partnership evaluation, sustainability plan, and partnership sustainability. All sub-components and their indicators were assessed to examine the strength of a cooperative–other actors’ partnerships. 

## 3. Methodology

### 3.1. Study Design

This study adopted the case study approach to provide a detailed description of a specific phenomenon within a given population [65]. According to Stake [66], the researchers can generalize the results of the case study for the future and across other settings. The intrinsic case study investigated in this paper was a partnership established by the Beekeepers Cooperative Association-Albaha in Saudi Arabia (hereafter, the BCA-A) with other actors. Partnerships were the unit of analysis in this paper. In this study, we used survey research to analyze a partnership’s characteristics from the viewpoint of the BCA-A. Therefore, the survey was administered to the executive director and employees of the BCA-A to collect data about the variables examined. A complete description was provided to highlight the context of the case study.

### 3.2. Case Description

Cooperative associations in Saudi Arabia were classified into nine categories: multi-purpose, agricultural, fishermen, beekeepers, housing, marketing, services, vocational, and consumer [67]. In 2020, the number of cooperative associations was 247 [68]. The BCA-A is located in the Al-Baha region in the southwestern part of the kingdom of Saudi Arabia. It was established on 9 February 2008 as the country’s first beekeepers’ cooperative association. The BCA-A is one of ten beekeeper cooperatives in Saudi Arabia. The number of general assembly members reached 154 beekeepers in 2022 [69]. The objectives of the BCA-A were creating a spirit of cooperation and coordination among members to serve their interests, representing a regulatory reference for beekeepers in the region, training and supervising beekeepers, and providing beekeeping services at reasonable prices. They also include developing marketing processes for the honey and its byproducts, enhancing honey bees’ nutritional and therapeutic importance and the economic value of their products, protecting and developing pasture lands, and representing members before the relevant authorities [69]. The strategy of the BCA-A from 2020 to 2025 includes establishing a link between beekeepers, government institutions, and civil society organizations and establishing partnerships with many local and international institutions. Additional strategies cover holding consultative meetings and organizing seminars and workshops on various apicultural topics, promoting community participation among beekeepers for sustainable rural communities, solving beekeepers’ problems by encouraging cooperation between beekeepers, and promoting social solidarity among beekeepers during natural disasters relating to bees [13].

A set of criteria guided the selection of the BCA-A for the current study: (a) One of the most successful cooperatives in attracting actors to work together in partnership at the national level during the investigation period (2016–2020); (b) The approval of the board of directors to conduct this study; (c) The cooperation with the study’s team to provide official partnership documents and address the questions accurately.

### 3.3. Sample

All partnerships between the BCA-A and other actors from 2016 to 2021 were selected (*n* = 33). Table 1 illustrates the distribution of these partnerships according to their establishment year. The complete description of each partnership (name of the partner, actor type, purpose of each partnership, and the partner’s website/social media account) was provided (supplementary material).

### 3.4. Data Collection Instrument

The variables of the data collection tool were developed based on a literature review highlighting partnerships between non-profit organizations and development actors [17,19,22,28,32,33,38,41,60,70]. A semi-structured questionnaire was developed to collect data from each partnership. Panel experts (*n* = 5) in agricultural cooperatives at King Saud University were invited to review the instrument’s questions to ensure reliability. Moreover, before conducting the study, ethics approval was obtained (Ref# HEC 22/242) from the Human Ethics Committee of King Saud University.

The questionnaire covered six criteria for analyzing a partnership. The first criterion included motivations for a partnership between the BCA-A and other actors. Then, the second criterion focused on the planning process of a partnership. It was divided into seven sub-criteria: partner, legal form, timescale, geographical coverage, purpose, objective, and type of partnership. The third criterion (outputs) comprised two sub-criteria, including stakeholders and services provided by a partnership. Governance practices were highlighted in the fourth criterion concerning managing, maintaining, reviewing, and revising them. Analyzing the outcomes of a partnership targeted in the fifth criterion highlighted the benefits gained from a partnership and defined how the impact of each partnership was measured. Finally, the sixth criterion summarized how a partnership was sustained by asking questions about the implementation level of sustaining outcomes practices and the current situation of a partnership. Altogether, the analytical framework for studying the partnerships between the BCA-A and other actors was divided into 15 sub-criteria with six main criteria, as depicted in Appendix A (Table A1).

Data were collected via face-to-face interviews with the executive director and some employees of the BCA-A during the period of July to August 2022. Additionally, content analysis was undertaken for the BCA-A’s website, partnership agreements, annexes, and official partnership documents to obtain the needed information according to the criteria and sub-criteria examined.

### 3.5. Variable Measurement and Data Analysis

The respondents were asked to determine the characteristics of each partnership according to the items examined in the 1st column of Table A1 in Appendix A. They selected all applicable from the data options list provided in the data collection tool. The questionnaire allowed them to add other options regarding each item to reflect the context of each partnership. A list of data options was coded, as illustrated in the 3rd column of Table A1, Appendix A. The final list of options was reviewed and presented in the results section. A focus group discussion (FGD) was held in September 2022 at King Saud University to discuss the suggested model for measuring the strength of the partnerships. Eight participants were invited to attend the meeting: the head of the board of directors of the BCA-A, the executive director of the BCA-A, two experts in agricultural economics, and four heads of the board of directors of beekeepers’ cooperative associations. The last author moderated the FGD to manage the discussions and summarize the views of the attendee regarding four areas. First, the appropriate weight value of each criterion regarding its influence on the strength of a partnership (2nd column, Table A1, Appendix A); second, points given for each option of data (4th column, Table A1, Appendix A); third, the minimum and maximum score points for each criterion and sub-criterion (5th column, Table A1, Appendix A), and finally, the calculation method of the partnership’s strength (6th column, Table A1, Appendix A).

During discussions, the FGD members were informed that the maximum point for the responses of the data options for each criterion and sub-criterion was based on the fact that they were asked to determine the weight point of each option depending on a five-point scale (0.2, 0.4, 0.6, 0.8, and 1). For example, if only one motivation existed for a partnership (1st row, Table A1, Appendix A), the FGD members decided to assign a point of (0.2) for this selection, 0.4 for two, 0.6 for three, and 0.8 for four motivations. However, 1 would be given if more than four motivations were present for a partnership. Likewise, the rating point for the other criteria and sub-criteria was clarified, as depicted in the 4th column of Table A1, Appendix A. Furthermore, the FGD members were asked to determine the weight of each criterion on a scale ranging from 1 to 5. Accordingly, the criterion of motivations, partnership planning, outputs, governance practices, outcomes, and sustainability of a partnership was assigned scores of 2, 4, 4, 3, 4, and 5, respectively (2nd column, Table A1, Appendix A).

The strength of each criterion was calculated using the Total Score Index (TSI):

TSI = ∑Pi × Wi(1)

where Pi = Each criterion point (4th column, Table A1, Appendix A), and Wi = Each criterion weight (weight given to each criterion provided by the respondents, ranging from 1 to 5).

In the case of the existence of a sub-criterion, the summed scores of all sub-criterions were calculated and then multiplied by the criteria’s weight (Wi) to calculate the TSI. The TSI scores of the six criteria were summated to determine the strength of each partnership. Accordingly, a partnership’s strength score was between a minimum of 16.2 and a maximum of 62. The partnership’s strength score was converted into a percentage and classified into three categories: low (<50%), moderate (50%–75%), and high (>75%). All information included in this paper was indexed, charted, and tabulated using frequencies and percentages.

## 4. Results

### 4.1. Analysis of the Characteristics of the Partnerships

#### 4.1.1. Motivations for Partnerships

Table 2 presents the findings of the organizational factors motivating the BCA-A to engage in partnerships with other actors. The findings revealed that the most crucial internal factors explaining why the BCA-A decided to engage in partnerships were achieving organizational goals (78.8%) and providing effective beekeeping services (78.8%). The results also revealed that enhancing financial stability was another critical motivator, mentioned in 63.6% of the partnerships established. Other factors reported that in more than a third of the partnerships investigated include development and innovation (42.2%), enhancing access to communities and stakeholders (39.4%), improving reputation and credibility (36.4%), and enhancing access to knowledge and expertise (36.4%).

#### 4.1.2. Planning of the Partnerships

Table 3 presents the distribution of the partnerships according to the partner-selection criteria applied by the BCA-A, where one can observe that the BCA-A mentioned multiple criteria for each partnership. Table 3 shows that the partner’s reputation and service quality were the most frequent selection criteria, with a percentage of 60.6%. Other criteria applied by the BCA-A in more than half of the partnerships investigated were to select the partners, including the statutory body (57.6%), and the importance of a partnership to the cooperative’s strategic plan (54.5%).

As part of the analysis of partnership planning, partnerships were examined to identify the form of institutional collaboration between actors, as illustrated in Table 4. The results indicated that the collaboration between the BCA-A and other cooperatives or not-for-profit organizations was observed in 12.1% of the total partnerships analyzed. In comparison, cross-sector partnerships were observed in the remaining partnerships (87.9%). Regarding the nationality of partners, the results revealed that the cooperative established partnerships with partners on various levels. However, among the partnerships analyzed, the national level of partners was observed in more than half of these collaborations (51.5%). Furthermore, the results in Table 4 reported the multiplicity of actors engaged with the cooperative in partnerships. Most collaborations were established with the private sector, with a percentage of 45.5%.

The formal arrangements between partners were also examined as part of the analysis of partnership planning. In this sense, a formal contract was regarded as the most critical legal arrangement between the BCA-A and other actors, seen in 48.5% of the partnership (Table 5). Nonetheless, some partners, particularly private sector partners, do not prefer legally enforceable agreements. Therefore, other friendlier legal arrangements (Memorandum of Understanding) were observed in 30.3% of the partnerships. However, one remarkable result found that informal agreements were established between partners based on a letter of association, noticed in 21.2% of the partnerships investigated.

Table 6 demonstrates the distribution of the partnerships according to the duration of the collaboration, where one can observe that the timescale varied across the partnerships analyzed. The findings indicated that the most popular partnership period was two years. This duration was observed in 39.4% of the partnerships. The next most popular duration for the collaborations was one year, seen in approximately one third of the partnerships (33.3%). In addition, less than one year and three years were also observed in 15.1% and 12.1% of the partnerships, respectively.

Partnerships were examined to determine their geographical coverage as part of the partnership analysis (Table 7). The findings depicted that more than half of them (51.5%) included activities in some regions. Moreover, 36.4% of the partnerships cover one region. Nevertheless, the percentage of partnerships covering various areas in one governorate or more reached 12.1% of the total partnerships established.

For the partnerships provided in Table 8, the findings revealed that the purpose of the collaboration multiplied across the partnerships implemented. One can notice that the honey value chain development was the most frequent reason attracting the partners to engage in the partnerships, with a percentage of 69.7%. The results also highlighted the importance of co-development of business manifested as one of the primary purposes in less than two-thirds of the partnerships (63.6%). In descending order, other purposes for collaboration included providing consultancy and extension services (45.5%), innovation and technology transfer (45.5%), and beekeeping market infrastructure development (30.3%).

Identifying the partnerships’ objectives is crucial to highlighting how the partnerships are planned (Table 9). The results indicated that developing and protecting pasture lands for bees and increasing productivity was the most frequent objective observed in most partnerships (69.7%). Furthermore, providing post-harvest handling practices was observed in approximately two-thirds of partnerships (66.7%). From the partners’ side, it was noticed that increased recognition of their role in social responsibility was the most beneficial in 45.1% of the corporations. It was also observed that the issues of obtaining direct funds and event sponsorships were among the objectives attracting actors to engage in partnerships with the cooperative.

The partnership types regarding the degree of social orientation versus business were examined to identify how each partnership is planned (Table 10). The overwhelming of partnerships could be characterized as strategic partnerships (87.9%), whereas the remaining partnerships could be labeled transactional. The semi-strategic partnerships (new commercial initiatives) where all partners could achieve their individual goals jointly, were observed in more than half of the partnerships (54.5%). Interestingly, the findings demonstrated that the BCA-A was jointly working with other partners to achieve a mutual benefit (core-business partnerships), seen in 33.3% of the partnerships. New commercial partnerships were the most frequent strategic partnerships (44.9%). In contrast, 14.5% of the strategic partnerships could be labeled core-business partnerships. Furthermore, the interaction of the cooperative and the partners for exchanging services or goods based on the size of sales and utilizing the partner’s products (social investment partnerships) was discernible in 12.1% of the partnerships researched.

#### 4.1.3. Outputs

**Figure 2:**
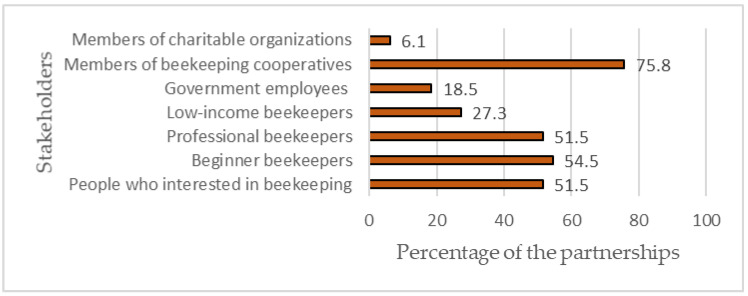
Stakeholders of the partnerships.

Figure 2 depicts the stakeholders targeted from the activities conducted within the partnerships examined. The findings confirmed that the partnerships researched targeted various clusters of beekeepers regarding their characteristics, including members of beekeeping cooperatives (75.8%), professional beekeepers (51.5%), beginner beekeepers (54.5%), people interested in beekeeping (51.5%), and low-income beekeepers (27.3%). Meanwhile, other stakeholders targeted by the partnerships included members of charitable organizations and government employees.

**Figure 3:**
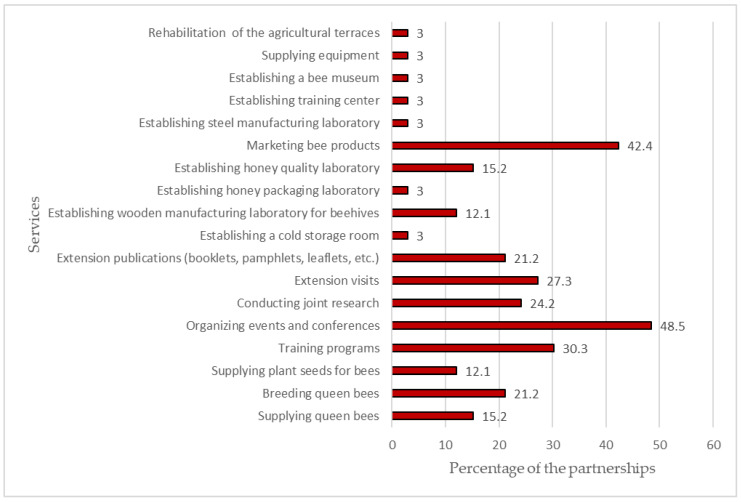
Services provided by the partnerships.

Some collaborations were delineated in specific activities, and others were involved in various services to underline the activities and services provided by the partnerships analyzed. Figure 3 denotes that the most frequent services were organizing events and conferences, marketing bee products, and conducting training programs, with percentages of 48.5%, 42.5%, and 30.3%, respectively. In the same context, partnerships targeted some services for boosting productivity and promoting the quality of bee products, such as supplying queen bees, breeding queen bees, supplying plant seeds for bees, and conducting joint research. Moreover, the development of beekeepers’ competencies through extension visits and preparing extension publications (booklets, pamphlets, etc.) was also noticed. Among other activities, services for building an asset for the BCA-A were considered valuable for the sustainability of the BCA-A in providing effective services. These services included establishing a cold storage room, a wooden manufacturing laboratory for beehives, a honey-packaging laboratory, a honey quality laboratory, a steel-manufacturing laboratory, a training center, a bee museum, and equipment. Finally, the role of the cooperative in community sustainability was observed by its partnership with the MALR in rehabilitating agricultural terraces.

#### 4.1.4. Governance Practices

**Figure 4:**
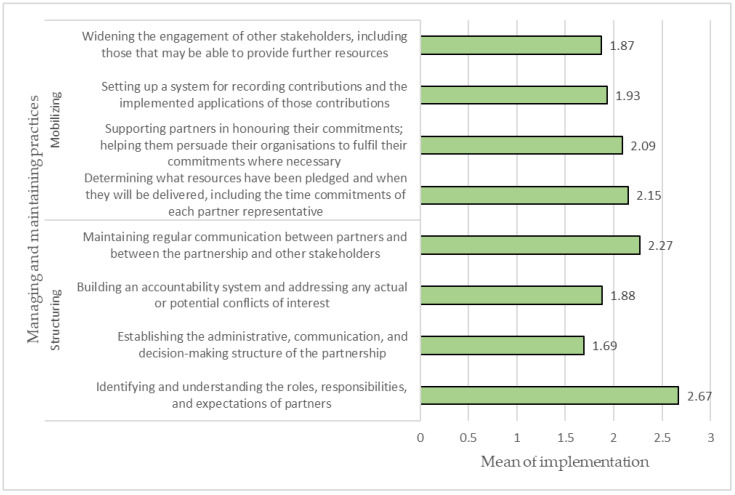
Managing and maintaining practices applied in the partnerships.

Figure 4 presents the implementation of managing and maintaining practices in the partnerships analyzed. The results reported that two items were considered to have a high level of implementation (mean > 2.25), while the remaining had a moderate level of implementation 1.5 > mean < 2.25. The practice of “maintaining regular communication between partners” was ranked first concerning implementation level, with a mean value of 2.27. In contrast, the item “establishing structure system of the partnership” was the least implemented practice in the partnerships surveyed, with a mean value of 1.69.

**Figure 5:**
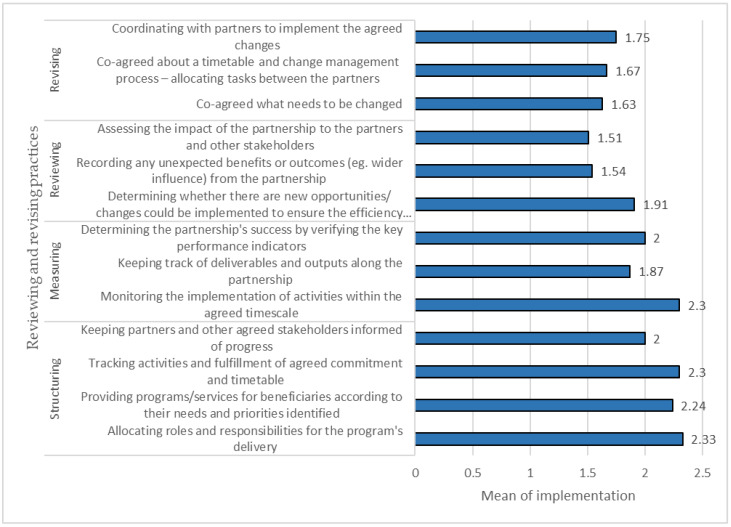
Reviewing and revising practices applied in the partnerships.

For reviewing and revising practices, Figure 5 presents the implementation means of these practices during the life cycle of the partnerships examined. As depicted in Figure 4, the implementation level ranged from low to moderate. The cooperative rated “allocating roles and responsibility for the program’s delivery” as having the highest level of implementation (mean = 2.33), while “assessing the impact of the partnership” with the lowest level of implementation (mean = 1.51).

#### 4.1.5. Outcomes

The partnerships were examined according to whether a specific methodology was applied for measuring the outcomes, as illustrated in Table 11. The results indicated that the following tools or frameworks evaluated only 6.1% of the collaborations. However, these partnerships were evaluated simply by assessing stakeholder satisfaction.

Table 12 demonstrates that effective beekeeping services (81.8%) were the most frequent benefit from the BCA-A’s point of view concerning the outcome gained for the cooperative resulting from engagement in partnerships. Furthermore, increased access to financial capital (72.7%) and solving beekeepers’ problems (69.7%) were critical benefits in more than two thirds of the partnerships examined. Other benefits included human capital development, gaining expertise from the partners, enhanced reputation, and organizational innovation.

#### 4.1.6. Sustainability of the Partnerships

**Figure 6:**
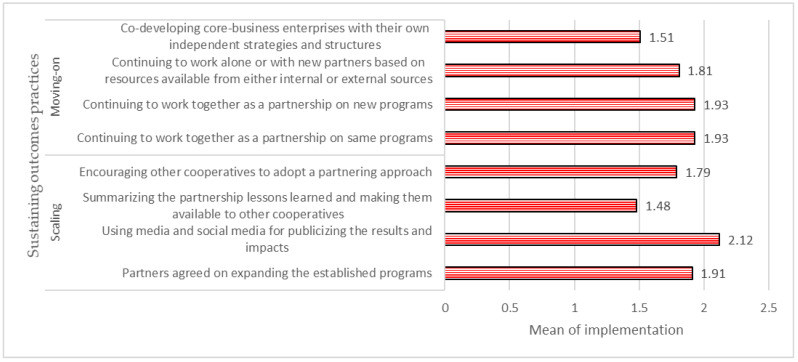
Sustaining outcomes practices applied in the partnerships.

The implementation level of seven statements assessed in scaling and moving practices was considered moderate, while one was considered a high-level implementation regarding sustaining outcomes (Figure 6). As in Figure 5, the highest implementation level was observed for “using media and social media for publicizing the results of the partnerships”, with a mean of 2.12. On the contrary, the item “summarizing the lessons learned of the partnerships” was the least implemented practice with a mean of 1.48.

**Figure 7:**
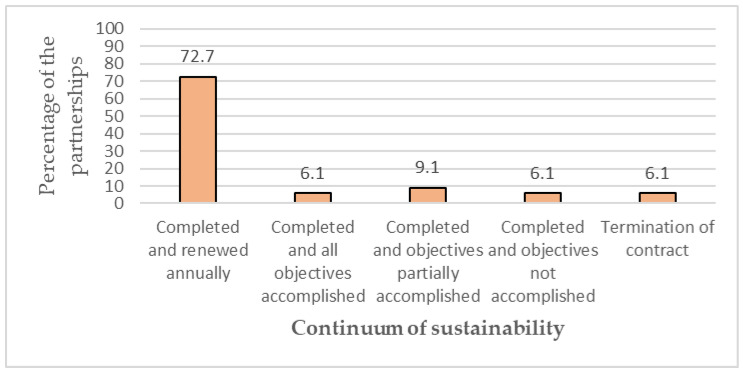
Sustainability continuum of the partnerships.

As part of analyzing the sustainability of the partnerships examined, Figure 7 highlights the situation after completing the collaborations between the BCA-A and other actors. Surprisingly, most collaborations (72.7%) were annually renewed, indicating the sustainability of a partnership. However, 9.1% attained their objectives partially during a partnership. Nevertheless, 6.1% of the partnerships accomplished all the planned goals. In this regard, one of the results deserving attention is that in two partnerships (6.1%) that were completed, objectives were not accomplished. Contrarily, two partnerships (6.1%) were terminated due to not obtaining the cooperative’s confidence.

### 4.2. Strength of the Partnerships

Table 13 illustrates the distribution of the partnerships according to their strengths. Per motivations, the values of a partnership’s strength ranged from 0.4 to 2 (Table 13), with an overall mean average of 1.46, indicating a high level of availability from the BCA-A’s perspective. Of the 33 partnerships investigated, 22 (66.66%) received the maximum score (2) for the motivations’ criteria. Furthermore, Table 13 reveals that the BCA-A were moderately satisfied with the planning of the partnerships established, with an overall mean value of 19.38. Regarding the outputs, most partnerships scarcely achieved the planned outputs based on an overall mean score of 5.60. One can notice that the means of governance practices varied across the partnerships. Only one (P32) achieved a high level of governance (mean ≥ 6). Likewise, the means of outcomes differed remarkably among partnerships, not attaining high levels. Table 13 depicts that six partnerships (18.2%) were considered to have a high level of sustainability (mean > 7.5). Three partnerships (P10, P19, and P33) were considered to have a high level of strength between partners, with 9.1% of the total partnerships investigated. On the contrary, 22 partnerships (66.66%) could be characterized with moderate strength and eight (24.23%) with a low one. The total partnerships’ strength score ranged from 41.81 with Asra for local agricultural products (P26) to 83.04 for the partnership with Saudi Arabian Oil Co (P10). 

## 5. Discussion

Previous studies suggested the role of cross-sector partnerships in cooperative sustainability. However, only a few empirical studies have systematically assessed the level of partnership strength and the features impacting outcomes and the organizational effectiveness of cooperatives. This paper contributes to the literature by analyzing the characteristics and strength of agricultural cooperatives–other actors’ engagements for sustainable rural development. It contributes to the existing literature and a conceptual framework as we quantify the strength of 33 partnerships signed between the BCA-A and the public or private actors during the period examined.

The impressive number of partnerships signed between 2016 and 2021 between the BCA-A and other actors was reported in this study. This result indicates the role of Saudi Vision 2030 in supporting partnerships between cooperatives and development actors. This conclusion was supported by the results of Alotaibi and Kassem [17], who found that Saudi Vision 2030 developed a conducive environment for encouraging the private sector to partner with agricultural cooperatives. Saudi Vision 2030 allowed cooperatives to act as business enterprises to implement projects funded by public agencies. In this regard, the Ministry of Human Resources and Social Development launched a new program for the cooperative sector, “Development of Cooperative Societies”, in 2019 [71]. One of this program’s main objectives was to develop standards for partnerships facilitating the construction of explicit business models for cooperative sustainability.

The results also revealed that most partnerships were motivated by achieving organizational objectives. This result supports the key concept of systems theory; the whole is greater than the sum of its parts [72]. According to Graikioti et al. [73], solving many constraints faced by cooperatives needs many capabilities and resources that cannot be fulfilled separately. Among other motivations for partnerships, acquiring financial resources is one of the most crucial motives for the cooperative to partner. As Candemir, Duvaleix and Latruffe [13] indicated, cooperatives aim for legitimacy and business growth opportunities, and this process cannot be achieved without financial stability. Therefore, this orientation forces cooperatives to find new funding sources to achieve their strategic plans [24].

Interestingly, the results suggested that partners aim to provide environmental services to society by engaging with the BCA-A. Many partnerships are targeted at developing and protecting pasture lands for bees. This objective is considered two-fold: one serves the interests of the cooperative’s members and the other for natural resource management. The availability of bee habitats mainly determines sustainable beekeeping. Planting or encouraging more-or-less permanent bee pastures is critical in increasing bee populations and improving bee nutrition [74]. However, enhancing bee habitats requires adopting good beekeeping practices, including proper grazing management (no overgrazing), diversification of pasture species, and management to increase the flowering period [75]. These practices are conducted within three types of bee pastures: single-year productive, multi-year productive, and permanent productive. Single-year productive bee pastures include wildflowers, ornamentals, and annual clovers collectively blooming for most of the season. However, multi-year productive bee pastures have plants that bloom all season, such as flowers, bushes, and some woody vines. Moreover, permanent productive bee pastures include trees, bushes, and a few woody perennials lasting for over 30 years to provide the most dependable source of nectars and pollens [76].

A weighted scoring model proposed in this study provides a reliable quantitative method for assessing the strength of a partnership. The ideal practices and weaknesses points are summarized based on the implementation level of criteria and sub-criteria included in the model. The results suggested that only three partnerships, one with the public and two with the private sector, were assessed to have a high level of strength. This result reflects that most partnerships examined still need to be enhanced in terms of features analyzed by the model, particularly governance practices and impact measurement. Concerning governance practices, the results revealed that the implementation level was still below the expected level in most partnerships. This result may be attributed to the fact that the BCA-A could not perform these practices effectively and efficiently. The reasons could include the nature of the issue to be addressed, a lack of required competencies, and the level of engagement with partners in performing activities. In this regard, international success stories of this issue emphasized the role of third parties as brokers for collaborative governance [77]. As mentioned by Stadtler and Probst [78], brokers can play three roles in overcoming good governance challenges. They can play the role of a converter to connect partners and help in conducting scale up approaches. They also moderate discussions and mediate between different interests. A learning catalyst role is also a crucial role for brokers to implement best practices of governance and monitoring and evaluation tools. Consequently, to enable brokers to manage a partnership process successfully, the broker should be aware, from the beginning, of the partnership, its role, and how willing it is to achieve the planned activities [77]. However, the findings illustrated that conducting partnership impact assessment is rarely used to measure the success of the collaborations. A probable explanation of this result may be the lack of knowledge and skill evaluation indicators, tools, or methodologies among the cooperative’s employees. Non-interest or lack of knowledge regarding the importance of evaluation between partners can be costly, mainly if a third party conducts it. This result is supported by Alotaibi and Kassem [17]. They found that only 17.2% of the partnerships established by different types of agricultural cooperatives and development actors in Saudi Arabia have been evaluated by pursuing tools or frameworks.

## 6. Conclusions

This paper addresses the characteristics of partnerships between agricultural cooperatives and development actors and their strengths based on a case study of the BCA-A in Saudi Arabia. This topic has been limitedly studied in the literature regarding the Saudi context. This study contributes to the literature because it is one of the first to suggest a weighted scoring model for the strength of partnerships. Eight conclusions are drawn based on the suggested conceptual framework and empirical investigation in this study. First, providing more effective beekeeping services, achieving the strategic plan, and leveraging financial resources have attracted and oriented the BCA-A to engage in partnerships. Second, partnerships established by the BCA have positively impacted honey value chain development, innovation, and technology transfer. Third, achieving environmental objectives has a particular relevance along with economic objectives. Fourth, the development of assets (buildings, laboratories, equipment, etc.) is targeted through the strategic partnership to ensure BCA-A’s sustainability. Fifth, limited evidence about the impacts of the partnerships on stakeholders and society is recognized due to not applying impact measurement studies in most partnerships investigated. Sixth, progress is attained in solving beekeepers’ problems, service excellence, and enhancing the BCA-A’s reputation resulting from the sustainable value creation of collaborations. Seventh, the continuity of most partnerships after the completion date reflects the trust of partners in the BCA-A, the quality of services provided, and the impact achieved at the cooperative and community levels. Eighth, designing partnerships must be enhanced in terms of the features examined in the proposed model to ensure their strength.

Analysis of the key features of the partnership and assessing their strength has both theoretical and practical implications. Concerning the theoretical implications, first, the conclusion further enriches the theory of partnerships by suggesting that partnerships can indeed strengthen cooperatives’ capacity to achieve their organizational objectives and that all partners gain mutual benefit. Second, the conclusion further supports the cooperative theory by verifying the critical role of agricultural cooperatives in innovation and technology transfer and providing many economic, social, and environmental services. Two practical implications can be concluded from this study. First, the importance of further popularizing partnerships in the cooperative sector occurs by developing multi-stakeholder platforms to articulate policy frameworks guiding public and private actors with the agricultural cooperatives. Second, the assessment of a partnership as successful should be supported by more rigorous methodologies systematically evaluating the outcomes of engagements.

On top of that, the suggested model for assessing a partnership’s strength offers a practical guide with a tested and reliable rating scale to orient future studies on this topic. Thanks to the suggested model, we inform partnerships about institutional weaknesses and offer guidance for developing the processes and practices needed to build truly collaborative partnerships performed at levels higher than the norm. This study has limitations because we have only focused on one cooperative without considering other agricultural cooperatives. Given the scarcity of research on partnership strength in the cooperative sector, further studies incorporating various types of cooperatives in different settings are necessary to test the external validity of our results. It may help improve our understanding of the strength of partnerships in a broader context. Additionally, on the one hand, incorporating economic impact indicators to assess the partnership value will enrich the proposed model. On the other hand, determining the economic value by the type and number of partners and the organizational capacity of cooperatives will enhance it further.

## Figures and Tables

**Table 1:** Distribution of the partnerships signed between the Beekeepers Cooperative Association-Al-Baha and other actors from 2016 to 2021.

Year	Number	%
2016	14	42.4
2017	4	12.1
2018	4	12.1
2019	5	15.2
2020	2	6.1
2021	4	12.1
Total	33	100

**Table 2:** Motivations of creating partnerships.

Drivers (*n* = 33)	Frequency	%
Achieving organizational goals	26	78.8
Developing human capital	10	30.3
Development and innovation	14	42.4
Enhancing financial stability	21	63.6
Providing effective beekeeping services	26	78.8
Improving reputation and credibility	12	36.4
Creating cooperative’s legitimacy	5	15.2
Enhancing access to communities and stakeholders	13	39.4
Enhancing access to knowledge and expertise	12	36.4
Purchasing services at preferable rates	5	15.2

**Table 3:** The partner-selection criteria applied by the cooperative.

Criteria (*n* = 33)	Frequency *	Percentage
Statutory body	19	57.6
Partner’s reputation	20	60.6
Partner’s financial stability or size of financial resources acquired	15	45.5
Strategic value for the future	14	42.4
Service quality (added value for stakeholders)	20	60.6
The importance of a partnership to the cooperative’s strategic plan	18	54.5
Previous experience with the partner	3	9.1

* Each item could be included in more than one partnership; percentages of the categories do not add up to 100%.

**Table 4:** Distribution of the partnerships according to the institutional form, nationality of the partners, and actor type.

Variable (*n* = 33)	Number	Percentage
Institutional form		
Intra-sector	4	12.1
Cross-sector	29	87.9
Nationality of partners		
Regional	5	15.1
National	17	51.5
International	11	33.3
Actor type		
Public sector	9	27.3
Private sector	15	45.5
University	2	6.1
Banks	2	6.1
Not-for-profit organizations	2	6.1
Cooperatives	2	6.1
UN organizations	1	3

**Table 5:** The legal form applied by the partnerships investigated.

Variable (*n* = 459)	Number	Percentage
Letter of association	7	21.2
Terms of reference (TOR)	-	-
Memorandum of Understanding (MoU)	10	30.3
contract	16	48.5

**Table 6:** The timescales of the partnerships established.

Variable (*n* = 33) *	Number	Percentage
<One year	5	15.1
One year	11	33.3
Two years	13	39.4
Three years	4	12.1

* A partnership’s duration in this table was determined based on the partnership’s annex at the beginning of the collaboration. Extending some partnerships to a specific period does not affect the process.

**Table 7:** The geographical coverage of the partnerships examined.

Variable (*n* = 33)	Number	Percentage
Governorate	3	9.1
Some governorates	1	3.0
Region	12	36.4
Some regions	17	51.5
National	-	-

**Table 8:** The purpose of the partnerships investigated.

Variable (*n* = 33)	Frequency *	Percentage
Development of honey value chain	23	69.7
Innovation and technology transfer	15	45.5
Beekeeping market infrastructure development	10	30.3
Co-development of business	21	63.6
Providing consultancy and extension services	15	45.5

* Each item could be included in more than one partnership; percentages of the categories do not add up to 100%.

**Table 9:** Objectives of the partnerships investigated.

Variable (*n* = 33)	Frequency *	Percentage
Developing and protecting pasture lands for bees	23	69.7
Increasing productivity	23	69.7
Enhancing quality (post-harvest handling practices)	22	66.7
Improving human resources skills of the cooperative’s employees	7	21.2
Recruitment	4	12.1
Direct funding	10	30.3
Indirect funding	5	15.2
In-kind subsidies	8	24.2
Producing byproducts of bees (bee pollen, propolis, bee bread, royal jelly, and beeswax)	8	24.2
Improving honey marketing channels	8	24.2
Event sponsorship	10	30.3

* Each item could be included in more than one partnership; percentages of the categories do not add up to 100%.

**Table 10:** Types of the partnerships.

Types (*n* = 33)	Number	%
A-Transactional partnerships		
Philanthropic	-	-
Social investments	4	12.1
B-Strategic partnerships		
New commercial initiatives	18	54.5
Core-business	11	33.4

**Table 11:** Distribution of partnerships according to use of evaluation methodology.

Variable (*n* = 33)	Number	%
Yes	2	6.1
No	31	93.9

**Table 12:** Outcomes of the partnerships.

Outcomes	Frequency *	%
Effective beekeeping services	27	81.8
Solving beekeepers’ problems	23	69.7
Human capital development	11	33.3
Gaining expertise from the partners	9	27.3
Increased access to financial capital	24	72.7
Enhanced reputation	20	60.6
Organizational innovation	16	48.5

* Each item could be included in more than one partnership; percentages of the categories do not add up to 100%.

**Table 13:** Total score for the strength of the partnerships.

Criteria	Total Score																	
	P1 *	P2	P3	P4	P5	P6	P7	P8	P9	P10	P11	P12	P13	P14	P15	P16	P17	P18
1. Drivers	2.00	0.40	1.2	2.0	1.6	2.0	2.0	0.40	0.40	2.00	1.20	2.00	2.00	2.00	1.60	2.00	2.00	2.00
2. Planning a partnership	20.4	16.0	17.4	23.0	22.8	23.2	16.8	18.4	14.8	26.4	22.0	20.2	22.2	19.0	14.8	17.8	23.8	20.4
2.1. Partner	3.60	3.20	3.80	3.80	3.60	3.20	3.20	3.20	2.80	4.00	3.60	3.40	3.80	3.00	2.80	3.40	3.80	3.60
2.2. Legal form	3.20	3.20	1.60	4.00	1.60	3.20	3.20	4.00	1.60	4.00	3.20	3.20	3.20	4.00	3.20	3.20	4.00	4.00
2.3. Timescale	1.60	2.40	2.40	3.20	4.00	4.00	1.60	2.40	1.60	4.00	3.20	3.20	3.20	1.60	1.60	2.40	2.40	2.40
2.4. Geographical coverage	2.40	2.40	3.20	3.20	3.20	3.20	0.80	3.20	2.40	3.20	3.20	2.40	3.20	2.40	2.40	2.40	2.40	2.40
2.5. Purpose	2.40	1.60	1.60	2.40	3.20	3.20	2.40	0.80	1.60	4.00	2.40	2.40	3.20	2.40	0.80	0.80	3.20	1.60
2.6. Objectives	4.00	0.80	2.40	4.00	3.20	3.20	2.40	0.80	1.60	3.20	3.20	2.40	1.60	2.40	0.80	2.40	4.00	2.40
2.7. Type of a partnership	3.20	2.40	2.40	2.40	4.00	3.20	3.20	4.00	3.20	4.00	3.20	3.20	4.00	3.20	3.20	3.20	4.00	4.00
3. Governance	5.92	5.09	5.11	5.34	5.27	4.31	4.48	2.23	2.71	5.39	5.80	3.92	2.70	3.31	3.37	3.79	2.68	4.19
3.1. Managing and maintaining	3.00	2.63	2.88	2.88	2.50	2.00	2.25	1.00	1.25	2.63	2.88	2.00	1.63	2.00	1.75	2.25	1.38	2.50
3.2. Reviewing and revising	2.92	2.46	2.23	2.46	2.77	2.31	2.23	1.23	1.46	2.77	2.92	1.92	1.08	1.31	1.62	1.54	1.31	1.69
4. Outputs	1.60	1.60	2.40	4.00	5.60	5.60	2.40	3.20	4.00	6.40	1.60	3.20	3.20	4.00	1.60	1.60	5.60	4.80
4.1. Stakeholders	0.80	0.80	1.60	2.40	3.20	4.00	1.60	2.40	2.40	2.40	0.80	1.60	2.40	3.20	0.80	0.80	4.00	4.00
4.2. Activities and services	0.80	0.80	0.80	1.60	2.40	1.60	0.80	0.80	1.60	4.00	0.80	1.60	0.80	0.80	0.80	0.80	1.60	0.80
5. Outcomes	2.40	2.40	1.60	3.20	3.20	2.40	2.40	4.80	2.40	4.80	2.40	1.60	3.20	1.60	1.60	2.40	3.20	3.20
5.1. Benefits	1.60	1.60	0.80	2.40	2.40	1.60	1.60	2.40	1.60	4.00	1.60	0.80	2.40	0.80	0.80	1.60	2.40	2.40
5.2. Impact measurement	0.80	0.80	0.80	0.80	0.80	0.80	0.80	2.40	0.80	0.80	0.80	0.80	0.80	0.80	0.80	0.80	0.80	0.80
6. Sustainability of a partnership	7.83	7.33	6.50	7.17	6.83	6.83	7.67	3.10	6.03	6.50	8.00	4.87	5.83	2.30	3.73	3.73	6.00	5.50
6.1. Sustainability plan	3.83	3.33	2.50	3.17	2.83	2.83	3.67	1.50	2.83	2.50	4.00	1.67	1.83	1.50	1.33	1.33	2.00	1.50
6.2. Current situation	4.00	4.00	4.00	4.00	4.00	4.00	4.00	1.60	3.20	4.00	4.00	3.20	4.00	0.80	2.40	2.40	4.00	4.00
Total	40.16	32.82	34.21	44.70	45.30	44.34	35.75	32.13	30.34	51.49	41.00	35.79	39.14	32.21	26.70	31.32	43.28	40.09
Percent	64.52	52.93	55.17	72.09	73.06	71.52	49.65	51.82	48.93	83.04	66.12	57.72	63.13	51.95	43.06	50.51	69.81	64.66
Level	M	M	M	M	M	M	L	M	L	H	M	M	M	M	L	M	M	M
**Criteria**	**Total Score**																**Mean**	**SD**
	**P19**		**P20**	**P21**	**P22**	**P23**	**P24**	**P25**	**P26**	**P27**	**P28**	**P29**	**P30**	**P31**	**P32**	**P33**		
1. Drivers	2.00		2.00	2.00	2.00	2.00	0.80	0.80	0.80	0.80	2.00	0.40	0.40	0.80	0.80	2.00	1.46	0.65
2. Planning a partnership	24.6		21.40	23.00	16.00	20.60	16.20	15.80	15.80	16.00	17.60	16.00	17.20	17.00	19.20	24.00	19.38	3.27
2.1. Partner	3.80		3.80	3.80	3.20	3.80	3.40	3.00	3.00	3.20	3.20	3.20	3.60	3.40	3.20	4.00	3.43	0.34
2.2. Legal form	4.00		4.00	4.00	3.20	4.00	4.00	4.00	4.00	4.00	1.60	1.60	1.60	1.60	4.00	4.00	3.24	0.93
2.3. Timescale	3.20		2.40	3.20	2.40	2.40	2.40	1.60	1.60	1.60	2.40	2.40	2.40	2.40	1.60	3.20	2.49	0.74
2.4. Geographical coverage	3.20		3.20	3.20	0.80	3.20	2.40	0.80	1.60	2.40	3.20	3.20	3.20	3.20	2.40	3.20	2.64	0.73
2.5. Purpose	4.00		3.20	2.40	1.60	1.60	0.80	1.60	0.80	0.80	1.60	0.80	.80	1.60	1.60	4.00	2.03	1.02
2.6. Objectives	3.20		0.80	2.40	1.60	2.40	0.80	1.60	1.60	0.80	1.60	1.60	1.60	1.60	3.20	1.60	2.15	0.98
2.7. Type of a partnership	3.20		4.00	4.00	3.20	3.20	2.40	3.20	3.20	3.20	4.00	3.20	4.00	3.20	3.20	4.00	3.36	0.51
3. Governance	2.88		2.63	4.67	5.47	5.80	3.12	2.93	3.18	3.24	3.07	2.00	2.54	3.98	6.00	4.95	4.00	1.22
3.1. Managing and maintaining	1.50		1.63	2.75	2.63	2.88	1.50	1.63	1.88	1.63	1.38	1.00	1.00	1.75	3.00	2.88	2.07	0.65
3.2. Reviewing and revising	1.38		1.00	1.92	2.85	2.92	1.62	1.31	1.31	1.62	1.69	1.00	1.54	2.23	3.00	2.08	1.93	0.63
4. Outputs	7.20		2.40	4.80	1.60	4.80	2.40	2.40	1.60	1.60	1.60	3.20	3.20	3.20	4.00	5.60	3.39	1.62
4.1. Stakeholders	4.00		0.80	4.00	0.80	4.00	0.80	0.80	0.80	0.80	0.80	2.40	2.40	2.40	2.40	4.00	2.13	1.24
4.2. Activities and services	3.20		1.60	0.80	0.80	0.80	1.60	1.60	0.80	0.80	0.80	0.80	0.80	0.80	1.60	1.60	1.26	0.74
5. Outcomes	4.00		4.00	4.00	3.20	2.40	2.40	1.60	1.60	1.60	3.20	3.20	4.00	4.80	1.60	4.80	2.88	1.05
5.1. Benefits	3.20		3.20	3.20	2.40	1.60	1.60	0.80	0.80	0.80	2.40	2.40	3.20	2.40	0.80	4.00	1.98	0.94
5.2. Impact measurement	0.80		0.80	0.80	0.80	0.80	0.80	0.80	0.80	0.80	0.80	0.80	0.80	2.40	0.80	0.80	0.89	0.38
6. Sustainability of a partnership	6.33		5.67	6.67	7.83	8.00	2.47	5.83	2.93	3.90	6.17	5.33	5.50	6.33	7.83	7.67	5.88	1.67
6.1. Sustainability plan	2.33		1.67	2.67	3.83	4.00	1.67	1.83	1.33	1.50	2.17	1.33	1.50	2.33	3.83	3.67	2.41	0.92
6.2. Current situation	4.00		4.00	4.00	4.00	4.00	0.80	4.00	1.60	2.40	4.00	4.00	4.00	4.00	4.00	4.00	3.46	0.99
Total	47.02		38.09	45.14	36.10	43.60	27.38	29.37	25.92	27.14	33.63	30.13	32.84	36.11	39.43	49.02		
Percent	75.83		61.43	72.82	58.22	70.32	44.16	47.37	41.81	43.77	54.24	48.59	52.96	58.24	63.59	79.06		
Level	H		M	M	M	M	L	L	L	L	M	L	M	M	M	H		

H (High); M (Moderate); L (Low); * (P) refers to the code name of each partnership as indicated in Table S1 (Supplementary file).

## Data Availability

All data are presented within the article.

## Supplementary Materials

The following supporting information can be downloaded at: https://www.mdpi.com/article/10.3390/ijerph20010364/s1, Table S1. List of organizations engaged with Beekeepers Cooperative Association-Al Baha in the partnerships from 2016 to 2021.

## Appendix A

**Table A1:** Weighted scoring model for examining strength of the partnerships.

Criteria	Weight	Coding	Points	Score		Total	
				Min.	Max.	Min.	Max.
1. Motivations	2	Yes (1); No (0) for each option	1 option (0.2) 2 options (0.4) 3 options (0.6) 4 options (0.8) More than 4 options (1)	0.2	1	0.4	2
2. Planning a partnership	4			2.5	7	10	28
2.1. Partner			Average score of sub-criterions of 2.1 (2.1.1,2.1.2,2.1.3, and 2.1.4)	0.7	1	2.8	4
2.1.1. Partner-selection criteria		Yes (1); No (0) for each option	1 option (0.2) 2 options (0.4) 3 options (0.6) 4 options (0.8) More than 4 options (1)	0.2	1	0.8	4
2.1.2. Nationality of partners		Yes (1); No (0) for each option	0.6 (regional)	0.6	1	2.4	4
			0.8 (national)				
			1 (international)				
2.1.3. Actor type		Yes (1); No (0) for each option	1 for any option	1	1	4	4
2.1.4. Institutional form		Yes (1); No (0) for each option	1 for any option	1	1	4	4
2.2. Legal form		Yes (1); No (0) for each option	1 (contract)	0.2	1	0.8	4
			0.8 (MoUs)				
			0.6 (TOR)				
			0.4 (letter of association)				
			0.2 (informal)				
2.3. Timescale		Yes (1); No (0) for each option	0.4 (undetermined)	0.4	1	1.6	4
			0.4 (one year)				
			0.6 (one year–two years)				
			0.8 (>two years–<three years)				
			1 (Three years)				
2.4. Geographical coverage		Yes (1); No (0) for each option	0.2 (Governorate)	0.2	1	0.8	4
			0.4 (Some governorates)				
			0.6 (Region)				
			0.8 (Some regions)				
			1 (National)				
2.5. Purpose		Yes (1); No (0) for each option	1 option (0.2) 2 options (0.4) 3 options (0.6) 4 options (0.8) More than 4 options (1)	0.2	1	0.8	4
2.6. Objectives		Yes (1); No (0) for each option	Each objective was assigned according to its relative weight to the BCA-A *	0.4 *	1 *	1.6	4
2.7. Type of a partnership		Yes (1); No (0) for each option	0.4 (Philanthropic)	0.4	1	1.6	4
			0.6 (Social investments)				
			0.8 (New commercial initiatives)				
			1 (Core-business)				
3. Outputs	4			0.4	2	1.6	8
3.1. Stakeholders		Yes (1); No (0) for each option	1 option (0.2) 2 options (0.4) 3 options (0.6) 4 options (0.8) More than 4 options (1)	0.2	1	0.8	4
3.2. Activities and services		Yes (1); No (0) for each option	Each objective was assigned according to its relative weight to the BCA-A **	0.2 **	1 **	0.8	4
4. Governance	3			0.4	2	2	6
4.1. Managing and maintaining practices		High (3); Moderate (2); Low (1) for each statement	Average score of the statements was calculated High (1) Moderate (0.6) Low (0.2)	0.2	1	1	3
4.2. Reviewing and revising practices				0.2	1	1	3
5. Outcomes	4			0.4	2	1.6	8
5.1. Benefits		Yes (1); No (0)	1 option (0.2) 2 options (0.4) 3 options (0.6) 4 options (0.8) More than 4 options (1)	0.2	1	0.8	4
5.2. Measurement of the impact of a partnership		Yes (1); No (0)	1	0.2	1	0.8	4
		Yes (1); No (0)	0.6				
		0	0.2				
6. Sustainability of a partnership	5			0.4	2	2	10
6.1. Sustainability plan		High (3); Moderate (2); Low (1)	Average score of the statements was calculated High (1) Moderate (0.6) Low (0.2)	0.2	1	1	5
6.2. Current situation of a partnership		Yes (1); No (0)	1 (Completed and renewed annually)	0.2	1	1	5
		Yes (1); No (0)	0.8 (Completed and all objectives accomplished)				
		Yes (1); No (0)	0.6 (Completed and objectives partially accomplished)				
		Yes (1); No (0)	0.4 (Completed and objectives not accomplished)				
		0	0.2 (Termination of contract)				
Total				17.6		62	

* The score of 0.4 was given to the objectives of recruitment and in-kind subsidies. The score of 0.6 was assigned to improving human resources skills of the cooperative’s employee, indirect funding, and event sponsorship. The score of 0.8 was given to producing byproducts of bees and improving honey marketing channels. The score of 1 was assigned to developing and protecting pasture lands, increasing productivity, enhancing quality, and direct funding. The points of objectives were summated for each partnership according the rating points mentioned and then classified into five categories as follows: <2, 2–4, 4.1–6,6.1–8, >8. There categories were assigned scores of 0.2, 0.4, 0.6, 0.8, and 1, respectively. ** The score of 0.4 was given to services of extension visits, establishing a bee museum, extension publications, training programs, and organizing events and conferences. The score of 0.6 was assigned to services of supplying queen bees, supplying plant seeds for bees, and rehabilitating the agricultural terraces. The score of 0.8 was given to services for conducting joint research, marketing bee products, and supplying equipment. The score of 1 was assigned to breeding queen bees, establishing a cold storage room, wooden manufacturing laboratory for beehives, honey packaging laboratory, honey quality laboratory, steel manufacturing laboratory, and establishing a training center. The points of services were summated for each partnership according to the rating points mentioned, and then classified into five categories as follows: <2, 2–4, 4.1–6,6.1–8, >8. There categories were assigned scores of 0.2, 0.4, 0.6, 0.8, and 1, respectively.
