# Supplementary material for: A Framework for Quantifying the Strength of Partnerships between Agricultural Cooperatives and Development Actors: A Case Study in Saudi Arabia
Source: Int J Environ Res Public Health. 2022 Dec 26;20(1):364. doi: 10.3390/ijerph20010364 (PMC9819027; doi:10.3390/ijerph20010364)
Supplement: Supplementary file 1 [file ijerph-20-00364-s001.zip › ijerph-2062048-supplementary.pdf]

Table S1. List of organizations engaged with Beekeepers Cooperative Association-Al Baha in the partnerships from 2016 to 2021.

Code of Partnership	Organization	Type	Purpose of the Partnership	Website/Social Media Account
P1	Albaha Municipality	Public	- Logistic services.<br>- Event staffing.	https://amanatalbaha.gov.sa/
P2	Social Development Bank	Public bank	- Providing loans for the members of the cooperative.	https://www.sdb.gov.sa/en-us/our-products/personal/social-loans
P3	Saudi post / SPL	Public	- Marketing the bee products to the local clients.	https://splonline.com.sa/en/
P4	Cooperative Societies Council	Cooperative	- Funding projects.<br>- Providing consultancy services.	http://www.cscs.org.sa
P5	Eng. Abdullah Bugshan Chair for Bee Research	University	- Providing training and capacity building programs for the cooperative's beekeepers.<br>- Research.<br>- Extension services for the cooperative's beekeepers.	https://beechair.ksu.edu.sa/
P6	Technical and Vocational Training Corporation	Public	- Accreditation of the training programs provided by the cooperative.	https://tvtc.gov.sa/en/Pages/default.aspx

Code of Partnership	Organization	Type	Purpose of the Partnership	Website/Social Media Account
P7	Baljurashi Municipality	Public	- Logistic services.<br>- Event staffing.	https://sa.linkedin.com/company/baljurashi-municipality
P8	Eiffel Lebanon Honey World	Private	- Indirect funding by purchasing the products of the company (bee medicines and feed supplements).<br>- Consultancy services for the cooperative's beekeepers.	http://www.lebanonbeeworld.com/home/about
P9	Agricultural Cooperative Society In Baljurashi	Cooperative	- Providing training programs to the members of Agricultural Cooperative Society in Baljurashi.<br>- Extension services to the members of Agricultural Cooperative Society in Baljurashi.<br>- Establishing a bee pasture for the Agricultural Cooperative Society in Baljurashi.	https://ziraeihb.org.sa/
P10	Saudi Arabian Oil Co.	Private	- Funding projects.<br>- Capacity and building programs for the cooperative's beekeepers.<br>- Direct fund for the cooperative's beekeepers.<br>- Sponsorship of events.	https://www.aramco.com/
P11	Ministry of Tourism	Public	- Participating in events organized by the ministry.<br>- Supporting the role of the cooperative as a tourist destination.	https://mt.gov.sa/Pages/default.aspx

Code of Partnership	Organization	Type	Purpose of the Partnership	Website/Social Media Account
P12	The General Directorate of Prisons	Public	- Providing training and capacity building programs for inmates.	https://www.my.gov.sa/
P13	Al Najeh for Honey Bees & Bees Equipment	Private	- Supplying the cooperative with beekeeping supplies and equipment (indirect funding).	https://www.alnajeh.ae
P14	Diyar Alezz to Organize Events and Conferences	Private	- Organizing Events and Conferences	https://twitter.com/diyaralez1?lang=ar
P15	Three quarters to Organize Events and Conferences	Private	- Organizing Events and Conferences	
P16	Ministry of Human Resource and Social Development (MHRSD)	Public	- Funding projects.<br>- Youth entrepreneurship programs in the field of beekeeping.<br>- Training and capacity building programs.	https://hrsd.gov.sa/en
P17	Food and Agricultural Organization of the United Nations (FAO)	International United Nations Organization	- Funding projects.<br>- Consultancy services.<br>- Capacity building and training programs.	https://www.fao.org/home/en
P18	Agriculture Development Fund (ADF)	Public bank	- Loans for the cooperative's beekeepers.<br>- Funding projects.	https://adf.gov.sa/ar/Pages/default.aspx

Code of Partnership	Organization	Type	Purpose of the Partnership	Website/Social Media Account
P19	Ministry of Environment, Water, and Agriculture (MEWA)	Public	-Funding projects.	https://www.mewa.gov.sa/en/Pages/default.aspx
P20	Abu Dhabi Food Control Authority	Public	- Research, innovation and development of the honey bee sector<br>- Capacity building and training in the fields of beekeeping and beekeeping management.<br>- Cooperation in the field of disease diagnosis, laboratories, and treatment.<br>- Development of bee pastures and the exchange of seeds of pasture plants for bees.<br>- Queen bee development.<br>- Implementing appropriate training and extension programs for beekeepers.<br>- Implementing seasonal visits for beekeepers in the two countries.<br>- Organizing events and conferences.	https://www.adafsa.gov.ae/English/Pages/default.aspx
P21	Almajdouie Foundation	Not-for-profit	- Funding projects.<br>-Sponsorship of events.<br>- Providing consultancy services.	https://www.almajdouie.org/
P22	Al-Baha University	University	- Research.<br>- Organizing events and conferences.	https://bu.edu.sa/home

Code of Partnership	Organization	Type	Purpose of the Partnership	Website/Social Media Account
P23	Express Transportation Company Ltd.	Private	- Marketing bee products to the clients.	https://www.smsaexpress.com/sa/
P24	Lulu International Group	Private	- Marketing bee products in local hypermarkets of the company.	https://www.lulugroupinternational.com/
P25	AL SABLA Digital Solutions	Private	- e-marketing platform.	http://alsablasolutions.com/
P26	Asra for local agricultural products	Private	- e-marketing platform.	https://asra.sa/
P27	Arab Organization for Agricultural Development (AOAD)	Not-for-profit	- Organizing and providing training and capacity building programs.<br>- Consultancy services.	http://www.aoad.org/indexeng.htm
P28	River Wood Inc.	Private	- Design and manufacturing of Beekeepers' clothing.	https://riverwood.com.pk/

Code of Partnership	Organization	Type	Purpose of the Partnership	Website/Social Media Account
P29	Hive Alive	Private	- Providing feed supplements for honeybees to the cooperative (indirect funding).	https://hivealivebees.com/
P30	Al-Aseel Foundation for Honey and Bee Supplies	Private	- Providing beekeeping supplies and equipment to the cooperative (indirect funding).	https://asilbee.com/
P31	Wesam Albadyah for Organizing Celebrations and Festivals	Private	- Organizing events and conferences.	https://twitter.com/wesamalbadyah?lang=ar
P32	ANHB Group	Private	- Development of local queen bees.<br>- Marketing the products of each partner.<br>- Development of post-harvest practices of the cooperative's products.<br>- Providing training and capacity building programs for the beekeepers.<br>- Subsidizing the prices of beekeeping supplies for the beekeepers.	https://anhb.ae/
P33	Lyson Beekeeping	Private	- Providing beekeeping supplies and equipment to the cooperative (indirect funding).<br>- Consulting services.	https://www.lysonau.com.au/
